# Endovascular Recanalization for Symptomatic Subacute to Chronic Atherosclerotic Basilar Artery Occlusion

**DOI:** 10.3389/fneur.2019.01290

**Published:** 2019-12-13

**Authors:** Wei Zhao, Jinping Zhang, Yun Song, Lili Sun, Meimei Zheng, Hao Yin, Jun Zhang, Wei Wang, Ju Han

**Affiliations:** ^1^Department of Neurology, The First Affiliated Hospital of Shandong First Medical University, Jinan, China; ^2^Department of Neurology, Shandong Provincial Qianfoshan Hospital, Shandong University, Jinan, China

**Keywords:** atherosclerosis, basilar artery occlusion, endovascular treatment, delayed recanalization, stroke

## Abstract

**Background:** The prognosis is poor for patients with symptomatic subacute to chronic atherosclerotic basilar artery occlusion (BAO) refractory to medical therapy. There has been no consensus on the optimal treatment for these patients until now.

**Objectives:** To assess the feasibility of endovascular recanalization for patients with symptomatic subacute to chronic atherosclerotic BAO refractory to medical therapy.

**Methods:** Consecutive patients who underwent endovascular recanalization for symptomatic subacute to chronic symptomatic atherosclerotic BAO from May 2015 to October 2018 were enrolled in our stroke center. Demographic, clinical, angiographic, procedural, and follow-up data were collected and analyzed.

**Results:** Twenty-one patients were enrolled in this study [mean age 57.9 years; 90.5% male; median pretreatment National Institutes of Health Stroke Scale (NIHSS) score 10; median time from image-documented BAO to treatment 15 days]. The success rate of the procedure was 81.0% (17/21). Periprocedural perforator strokes occurred in two patients (9.5%, 2/21). At 90 days, there was one death due to pneumonia (unrelated to the procedure), and there were no recurrent cases of TIA or stroke in the other 16 patients. At 90 days, 76.5% (13/17) of patients achieved a good clinical outcome [mRS: modified Rankin Scale (mRS) scores 0–2], and 94.1% (16/17) of patients achieved an acceptable outcome (mRS scores 0–3). During the 17.4 ± 8.0-month clinical follow-up period, one patient suffered from Wallenberg syndrome at 29 months.

**Conclusions:** Our study suggests that endovascular recanalization for subacute to chronic symptomatic atherosclerotic BAO appears to be feasible in selected patients.

## Introduction

Acute basilar artery occlusion (BAO) is catastrophic with high morbidity and mortality (1, 2). A subset of patients survives the acute occlusion stage, and a substantial number of patients experience recurrent symptoms despite aggressive medical treatment. Recurrent infarctions in the brain stem and cerebellum can result in catastrophic outcomes for these patients (3). Presumably, the poor prognosis of subacute to chronic BAO hinges on hemodynamic compromise without adequate collateral circulation. Currently, there is no consensus on the optimal treatment for these patients.

Several randomized controlled trials (RCTs) have confirmed that rapid endovascular recanalization can improve functional outcomes and reduce mortality in patients with acute ischemic stroke with proximal vessel occlusion (4–6), but there is no consensus for whether endovascular treatment is feasible and safe for symptomatic subacute to chronic BAO.

The purpose of this study is to assess the feasibility of endovascular recanalization for patients with symptomatic subacute to chronic atherosclerotic BAO refractory to medical therapy.

## Materials and Methods

### Patient Enrollment

The Institutional Review Board of Qianfoshan Hospital approved the study. “Subacute to chronic occlusions” was defined as symptomatic (TIA or stroke) complete occlusion of an intracranial artery of presumed atherosclerotic etiology in whom endovascular therapy was performed beyond 48 h from the time last seen well (7). We retrospectively reviewed our prospective stroke intervention database for patients undergoing endovascular recanalization for symptomatic subacute and chronic atherosclerotic BAO from May 2015 to October 2018. All patients were unstable with progressive or fluctuating symptoms despite aggressive medical therapy. We considered aggressive medical management to include at least dual-antiplatelet therapy, statin therapy, optimized blood pressure and glucose control, as well as smoking cessation and an emphasis on healthy lifestyle.

**Figure 1:**
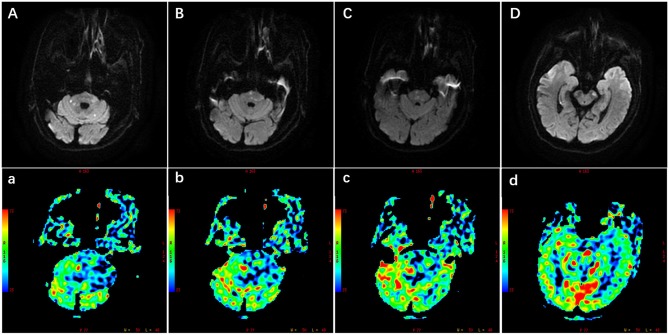
Illustration of multiple infarctions and perfusion defects in the posterior circulation, which were detected by the diffusion-weighted imaging (DWI) **(A–D)** and perfusion weighted imaging (PWI) **(a–d)** respectively. The PWI images were the cerebral blood flow images corresponding to the DWI images. Scale was color coded (red, largest cerebral blood flow; blue, least cerebral blood flow). PWI images showed larger perfusion deficits including the brain stem, cerebellum, and occipital lobe.

The common symptoms included dizziness, dysarthria, ataxia, hemiparesis, hemibody numbness, diplopia, memory disturbances, and so on, and the symptoms were exacerbated by activities. Multiple infarctions in brain stem, cerebellum, thalami, occipital lobe, and so on were detected by the brain magnetic resonance imaging (MRI). Hemodynamic failure was judged through computed tomography perfusion/magnetic resonance perfusion imaging, history, symptoms, or physical examination. Patients with other potential causes of BAO, such as vasculitis, arterial dissection, reversible cerebral vasoconstrictive syndrome, or embolic disease, were excluded. Dual antiplatelet treatment with 100 mg aspirin and 75 mg clopidogrel daily was routinely maintained for at least 5 days before the revascularization operation. BAO was initially assessed by non-invasive computed tomography angiography (CTA) or magnetic resonance angiography (MRA). The illustrative case of multiple infarctions and perfusion defects in posterior circulation is shown in Figure 1.

### Intervention Procedure

The occlusion course and the collateral circulation of the BA were evaluated comprehensively by digital subtraction angiography (DSA) before the endovascular recanalization procedures. All of the procedures were performed under general anesthesia. A 0.014 Synchro micro-guidewire (Stryker Neurovascular, USA), assisted by an Excelsior SL-10 soft microcatheter (Stryker Neurovascular, Ireland), was introduced into the occluded BA and carefully passed through the occluded lesion to the distal segment. After withdrawing the micro-guidewire, the length of the occlusion and distal lumen of the lesion were confirmed through the microcatheter angiography. We traversed the occluded segment and cannulated one of the two posterior cerebral arteries (PCAs) or the superior cerebellar arteries (SCAs). A distal access catheter (Concentric Medical, USA) was applied for those with an excessively tortuous vessel pathway. The lesions were initially predilated with conventional balloons (Gateway balloon, Boston Scientific, USA). The following application of the drug-coated balloon (DCB) (paclitaxel-coated coronary balloon, SeQuent Please, B. Braun, Germany) or stenting (Wingspan stent, Stryker Neurovascular, USA; Neuroform EZ stent, Stryker Neurovascular, USA; Apollo stent, Micro-port Neuro Tech, China; Solitaire AB stent, Medtronic, USA) depended on the discretion of the operator. The application method of the DCB was the same as described in our previous study (8). All patients or their authorized family members provided informed consent for the off-label use of the coronary DCB. Intravenous heparin boluses were given to maintain the activated clotting time between 250 and 300 s during the procedure. A low dose of tirofiban (Lunan Better Pharmaceutical Co., LTD., China), which is a glycoprotein IIb/IIIa antagonist with a short half-life, was used to prevent local platelet aggregation and early reocclusion. Intravenous tirofiban injection was administered at a low-dose bolus (0.25 mg/h) during the procedure. Additionally, intravenous tirofiban was continued at a rate of 0.15 mg/h for 12 to 24 h after the procedure. Antegrade flow through the previously occluded BA was graded using the Thrombolysis in Cerebral Ischemia (TICI) grading system. Technical success was determined by recanalization with a TICI grade ≥2b on postprocedural angiography. After the procedure, a cerebral computed tomography (CT) scan was performed immediately, the patients were transferred to the intensive care unit, and the blood pressure was strictly controlled. All patients were discharged on dual antiplatelets, consisting of aspirin and clopidogrel, which was maintained for 3 months for patients with only balloon angioplasty and 6 months for patients with stenting.

### Data Collection

Demographic, clinical, angiographic, procedural, and follow-up data were collected. The patients were scheduled to perform DSA at 3–6 months. Primary endpoints included successful recanalization (defined as TICI ≥2b) and good functional outcome (defined as mRS score ≤2 at 90 days). Secondary endpoints included symptomatic postprocedural TIA, stroke, intracranial hemorrhage, subarachnoid hemorrhage, and mortality.

### Statistical Analysis

Descriptive statistics were used in this study. Continuous data were expressed as the mean ± standard deviation (SD) or as the median with interquartile range (IQR). Categorical data were expressed as numbers and percentages. Statistical analysis was performed using SPSS version 19.0 for Windows (SPSS Inc., Chicago, Illinois, USA).

## Results

Twenty-one patients were enrolled in this study (Table 1), and the baseline clinical variables are summarized in Table 2. Hypertension (*N* = 18, 85.7%) and smoking (*N* = 9, 42.9%) were the most common risk factors. The mean age of the enrolled patients was 57.9 ± 8.6 years, with male predominance (19, 90.5%). The median pretreatment NIHSS score was 10 (IQR, 4–15), the median time from symptom onset to treatment was 27 days (range, 13–365 days; IQR, 17–47 days), and the median time from image-documented BAO to treatment was 15 days (range, 6–47days; IQR, 10.5–20 days).

**Table 1:** Clinical summary of 21 patients undergoing endovascular recanalization for basilar artery occlusion.

**Patient**	**Age (years)**	**Time between initial symptoms and treatment (days)**	**Time between image-documented BAO and treatment (days)**	**NIHSS score at admission**	**mRS score at admission**	**Successful recanalization (yes/no)**	**Post-procedural perfusion (TICI)**	**Complications**	**90-day mRS score**	**Recurrent TIAs or strokes**	**Restenosis**
1	70–75	365	6	5	4	No	NA	No	4	1	NA
2	65–70	27	20	21	5	Yes	3	No	NA	NA	NA
3	65–70	28	20	20	5	Yes	3	No	3	0	NA
4	60–65	27	18	15	4	Yes	3	No	1	0	NA
5	60–65	12	10	2	2	Yes	3	No	0	0	NA
6	55–60	17	17	2	2	No	NA	No	2	0	NA
7	55–60	47	47	11	4	Yes	3	No	2	0	No
8	55–60	26	21	23	5	Yes	3	No	3	0	No
9	55–60	21	18	10	4	Yes	3	No	1	0	Yes
10	45–50	50	42	16	4	No	NA	Dissection	3	0	NA
11	50–55	45	7	14	4	Yes	3	No	1	0	No
12	50–55	15	8	2	2	Yes	3	No	1	0	NA
13	50–55	365	12	7	3	Yes	3	No	0	0	NA
14	50–55	46	41	4	2	Yes	3	No	0	0	NA
15	45–50	14	7	4	2	Yes	3	Perforator ischemic stroke	3	0	NA
16	45–50	160	43	12	4	Yes	3	Perforator ischemic stroke	0	0	NA
17	45–50	13	13	16	4	No	NA	Dissection	3	0	NA
18	45–50	50	8	14	4	Yes	3	No	2	0	NA
19	45–50	18	15	4	2	Yes	3	No	0	1	No
20	70–75	16	13	8	3	Yes	3	No	0	0	NA
21	70–75	45	13	6	2	Yes	2b	No	0	0	NA

*BAO, basilar artery occlusion; NIHSS, National Institutes of Health Stroke Scale; mRS, modified Rankin scale; TICI, Thrombolysis in Cerebral Infarction; NA, not applicable*.

**Table 2:** Baseline clinical variables.

**Variables**	***N* (%)**	**Median (IQR)**
Age (years), mean (SD) y	57.9 ± 8.6	
Time between initial symptoms and treatment (days)		27 (17–47)
Time between image-documented BAO and treatment (days)		15 (IQR, 10.5–20).
Pretreatment NIHSS score		10 (4–15)
Male	19 (90.5)	
Hypertension	18 (85.7)	
Diabetes mellitus	5 (23.8)	
Hyperlipidemia	2 (9.5)	
Atrial fibrillation	0 (0)	
Coronary artery disease	3 (14.3)	
Smoking	9 (42.9)	
Technical success	17 (81.0)	
90-day mortality	1 (4.8)	
Periprocedural stroke	2 (9.5)	

*BAO, basilar artery occlusion; NIHSS, National Institutes of Health Stroke Scale; IQR, interquartile range*.

A total of 81.0% (17/21) of patients achieved successful recanalization. The treatment modalities and outcomes of the 17 patients are summarized in Table 3. Among the 17 successful patients, conventional balloon angioplasty was applied in three patients, conventional balloon angioplasty plus DCB angioplasty was applied in five patients, conventional balloon angioplasty plus stenting was applied in eight patients, and conventional balloon angioplasty plus DCB angioplasty and stenting was applied in one patient. Postrecanalization angiography demonstrated TICI grade 3 in 16 patients (94.1%, 16/17) and TICI grade 2b in one patient (5.9%, 1/17). The median residual stenosis was 20% (IQR, 15–25%).

**Table 3:** Treatment modalities and outcomes of 17 patients with successful endovascular recanalization.

**Variables**	***N* (%)**	**Median (IQR)**
CBA	3 (17.6)	
CBA + stenting	8 (47.1)	
CBA + DCBA	5 (29.4)	
CBA + DCBA + stenting	1 (5.9)	
TICI = 3	16 (94.1)	
TICI = 2b	1 (5.9)	
Residual stenosis (%)		20 (15–25)
Symptoms improved	15 (88.2%)	
90-day mRS score ≤2	13 (76.5)	
90-day mRS score ≤3	16 (94.1)	
90-day recurrent symptoms	0 (0)	

*CBA, conventional balloon angioplasty; DCBA, drug-coated balloon angioplasty; TICI, Thrombolysis in Cerebral Infarction; mRS, modified Rankin scale; IQR interquartile range*.

Four patients experienced failure of the procedure in our study: the guidewire could not traverse the occluded segment in two patients (patient 1 and patient 6), and stable antegrade flow could not be obtained in the other two patients (patient 10 and patient 17) due to dissection. Reperfusion was achieved in the two patients with dissection, but the antegrade flow was limited and then lost. Maybe the dissections were not very large, no obvious complications occurred in the two patients, and the cerebral CT after the procedure did not show obvious hemorrhage.

Periprocedural perforator ischemic strokes occurred in two patients (patient 15 and patient 16). Brain MRI showed new infarcts in the posterior circulation after the procedure in the two patients. Patient 16 showed aggravating ophthalmoplegia with a new minor brain stem infarct, and he was neurologically independent with conservative medical therapy before discharge. His mRS score was 0 at the 90-day follow-up. The other patient (patient 15) had a medullary infarction after the procedure; he experienced somnolence, left hemiplegia, dysarthria, and respiratory failure. He recovered gradually with medical and ventilator therapy, and his mRS score was 3 at the 90-day follow-up. Other periprocedural complications, such as intracranial hemorrhage, subarachnoid hemorrhage, reperfusion syndrome, or perforation, did not occur in this case series.

**Figure 2:**
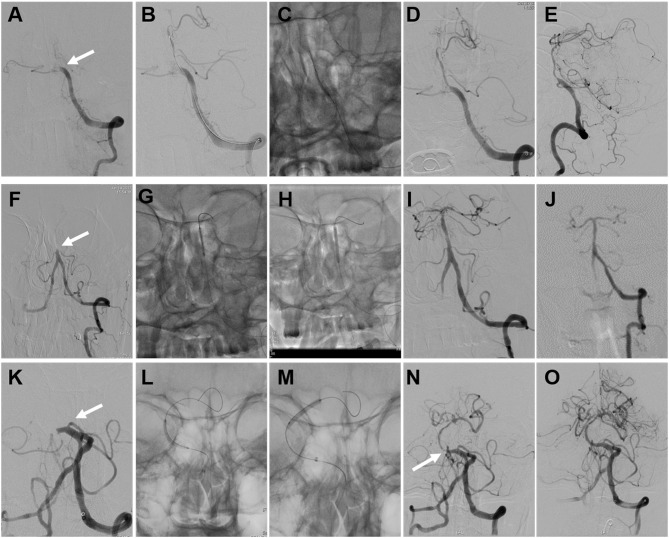
Illustrative cases of endovascular recanalization for subacute to chronic atherosclerotic basilar artery occlusion (BAO). Images **(A–E)** were of the angioplasty procedure for patient 18. **(A)** Total occlusion of the BA (arrow). **(B)** The guidewire traversed the occlusion site. **(C)** The angioplasty procedure with the conventional balloon. **(D,E)** Angiographic result after conventional balloon angioplasty. Images **(F–J)** were of the angioplasty procedure and follow-up digital subtraction angiography (DSA) data from patient 7. **(F)** Total occlusion of the BA (arrow). **(G)** The angioplasty procedure with the conventional balloon. **(H)** The angioplasty procedure with the drug-coated balloon (DCB). **(I)** Angiographic result after DCB angioplasty. **(J)** Angiographic result at the 4-month follow-up. Images **(K–O)** were of the angioplasty and stenting procedures for patient 16. **(K)** Total occlusion of the BA (arrow). **(L)** The angioplasty procedure with the conventional balloon. **(M)** The angioplasty procedure with the DCB. **(N)** Instant restenosis occurred at the initial segment of the BA after DCB angioplasty (arrow). **(O)** Angiographic result after remedial stenting.

We have continuous follow-up data for 17 successful cases. At 90 days, there was one death due to pneumonia (unrelated to the procedure), and there were no instances of recurrent TIA or stroke in the other 16 patients at the 90-day clinical follow-up. During the 17.4 ± 8.0-month clinical follow-up period, one patient (patient 19) suffered from Wallenberg syndrome at 29 months, which was considered unrelated to the procedure. The other 15 patients did not have recurrent TIAs or strokes. Symptoms improved in 15 patients, and the improvement rate was 88.2% (15/17). At 90 days, 76.5% (13/17) of patients achieved a good clinical outcome (mRS score 0–2), and 94.1% (16/17) achieved an acceptable outcome (mRS score 0–3). DSA follow-up was available for five patients, in-stent restenosis (≥50% stenosis) occurred in one patient (patient 9). The restenosis that occurred in patient 9 was asymptomatic with no recurrent symptoms. The average time of imaging follow-up was 9.8 ± 9.1 months. The remaining patients did not have any follow-up imaging. The illustrative cases of endovascular recanalization for subacute to chronic atherosclerotic BAO are shown in Figure 2.

## Discussion

The natural course of BAO is dismal, and the mortality rate is 85 to 95%, even with anticoagulant and fibrinolytic therapy, if the artery is not recanalized (1, 2); only 4% of cases of nonrecanalized BAO achieve good long-term outcomes (3). Many patients survive acute BAO and suffer from recurrent TIAs or progressive strokes, although with aggressive medical treatment. The prognosis of these patients is poor because of recurrent strokes in the posterior circulation. Presumably, inadequate collateral blood flow may play a role in symptom recurrence.

There has been no optimal treatment for these patients until now. The bypass procedure is technically challenging with high morbidity and mortality and has failed to show any benefit in the treatment of patients with medically refractory atherosclerotic occlusive disease and hemodynamic failure (9). Studies focusing on the endovascular recanalization of clinically unstable atherosclerotic subacute to chronic BAO are rare, and the enrolled patients were heterogeneous in terms of the etiology and occluded vessels (7, 10, 11). In the case series study of Dashti et al. (10) eight patients achieved successful endovascular recanalization for subacute and chronic BAO (88.9%, 8/9), four patients improved, four patients had periprocedural complications, and two died due to periprocedural complications. The high rates of periprocedural complications and mortality may be related to the small number and heterogeneity of patients.

To the best of our knowledge, our study represents the largest reported case series on endovascular recanalization for symptomatic subacute to chronic atherosclerotic BAO. Our study suggests that the procedure appears to be feasible in carefully selected patients. Except for one death during the follow-up period because of pneumonia and one patient who suffered from Wallenberg syndrome at 29 months, which were both unrelated to the procedure, the other 15 patients did not have recurrent TIAs or strokes during the 17.4 ± 8.0-month clinical follow-up period. The symptom improvement rate was 88.2% (15/17). At 90 days, the good clinical outcome (mRS score 0–2) rate was 76.5% (13/17), and the acceptable outcome rate was 94.1% (16/17). Although patient 3 and patient 8 had high pretreatment NIHSS scores (20 and 23, respectively), the symptoms improved after the procedure, and their mRS score was 3 at the last follow-up conversation.

The major technical challenge is traversing the occlusion site with a guidewire during recanalization. Prudent inspection of the CTA and the injection of both the internal carotid artery and vertebral artery during DSA are helpful for delineating the course of BAO and the collateral circulation. The development of interventional devices is also helpful for crossing occluded lesions. When opening chronic BAO, early reocclusion is also a high-risk concern. Tirofiban, a glycoprotein IIb/IIIa antagonist with a short half-life, has been used in some centers to prevent local platelet aggregation and early reocclusion during endovascular thrombectomy (12). Low-dose tirofiban has been indicated to improve functional outcomes in acute ischemic stroke patients treated with endovascular thrombectomy (13). Intra-arterial tissue plasminogen activator and abciximab (another type of glycoprotein IIb/IIIa antagonist) were used in two cases of subacute and chronic BAO endovascular recanalization when early reocclusion occurred in the previous study (10, 14). There were no cases of early reocclusion in our study, which might be a benefit of the application of low-dose tirofiban during and after the procedure in our center. Further trials are needed to investigate the efficiency and safety of the application of the glycoprotein IIb/IIIa antagonist in delayed BAO endovascular recanalization.

The procedure has high-risk potential because of periprocedural complications. The common complications include intracranial hemorrhage, subarachnoid hemorrhage, ischemic stroke, reperfusion syndrome, dissection, and perforation (7, 10, 11, 14, 15). The morbidity of perforator strokes may be high in delayed BAO recanalization due to the substantial number of perforator branches along the BA. It is critical to select patients who could benefit from this treatment.

Restenosis or in-stent restenosis is also an important concern during the procedure. DCB has been proven to effectively inhibit intimal hyperplasia and reduce the risk of restenosis in coronary and peripheral arteries (16, 17). Gruber et al. reported the application of DCB for intracranial symptomatic high-grade atherosclerotic stenosis and preliminarily demonstrated its superiority to stenting (18). Our previous study also demonstrated that the use of DCB may be feasible and safe for the treatment of symptomatic severe intracranial atherosclerotic disease (8). Six patients underwent DCB angioplasty in this study, and we need further studies and follow-ups to confirm whether DCB application is effective in reducing the restenosis rate in delayed BAO recanalization.

## Conclusions

This study proves that endovascular recanalization for symptomatic subacute to chronic atherosclerotic BAO may be feasible in carefully selected patients. But the procedure is not very safe and needs to be done in a very selective group of patients with great caution and has to be done in centers with adequate endovascular experience. The number of enrolled patients was not large enough, and angiographic follow-up data were lacking in most patients. The patient selection should be based on larger trials with adequate number of samples in each of the various endovascular technique arms described in the study, and the role of DCB for this indication needs further validation in larger highly selective population groups. The inherent flaws of a retrospective study along with a lack of a control arm were both limitations in this study. Further prospective randomized studies with larger patient numbers are needed to investigate the clinical efficacy and indications of the procedure.

## Data Availability Statement

The datasets generated for this study are available on request to the corresponding author.

## Ethics Statement

This study was carried out in accordance with the recommendations of the Institutional Review Board of Qianfoshan Hospital affiliated to Shandong University with written informed consent from all subjects. All subjects gave written informed consent in accordance with the Declaration of Helsinki. The protocol was approved by the Institutional Review Board of Qianfoshan Hospital affiliated to Shandong University.

## Author Contributions

WZ contributed to the design of the study, the acquisition, analysis, and interpretation of the data, drafting the article and revising the content, and final approval of the paper. JiZ, YS, LS, and MZ contributed to the analysis and interpretation of the data and the final approval of the article. HY, JuZ, and WW contributed to the statistical analysis, the revision of content, and the final approval of the article. JH contributed to the conception and design of the study, acquisition of the data, drafting of the article, revision of the content, and final approval of the article.

### Conflict of Interest

The authors declare that the research was conducted in the absence of any commercial or financial relationships that could be construed as a potential conflict of interest.

## References

[1] Hacke W Zeumer H Ferbert A Bruckmann H del Zoppo GJ. Intra-arterial thrombolytic therapy improves outcome in patients with acute vertebrobasilar occlusive disease. Stroke. (1988) 19:1216–22. 10.1161/01.STR.19.10.1216 3176080

[2] Brandt T von Kummer R Muller-Kuppers M Hacke W. Thrombolytic therapy of acute basilar artery occlusion. Variables affecting recanalization and outcome. Stroke. (1996) 27:875–81. 10.1161/01.STR.27.5.875 8623107

[3] Lindsberg PJ Soinne L Tatlisumak T Roine RO Kallela M Happola O. Long-term outcome after intravenous thrombolysis of basilar artery occlusion. JAMA. (2004) 292:1862–6. 10.1001/jama.292.15.1862 15494584

[4] Berkhemer OA Fransen PS Beumer D van den Berg LA Lingsma HF Yoo AJ. A randomized trial of intraarterial treatment for acute ischemic stroke. N Engl J Med. (2015) 372:11–20. 10.1056/NEJMoa1411587 25517348

[5] Campbell BC Mitchell PJ Kleinig TJ Dewey HM Churilov L Yassi N. Endovascular therapy for ischemic stroke with perfusion-imaging selection. N Engl J Med. (2015) 372:1009–18. 10.1056/NEJMoa1414792 25671797

[6] Goyal M Demchuk AM Menon BK Eesa M Rempel JL Thornton J. Randomized assessment of rapid endovascular treatment of ischemic stroke. N Engl J Med. (2015) 372:1019–30. 10.1056/NEJMoa1414905 25671798

[7] Aghaebrahim A Jovin T Jadhav AP Noorian A Gupta R Nogueira RG. Endovascular recanalization of complete subacute to chronic atherosclerotic occlusions of intracranial arteries. J Neurointervent Surg. (2014) 6:645–8. 10.1136/neurintsurg-2013-010842 24249733

[8] Han J Zhang J Zhang X Zhang J Song Y Zhao W. Drug-coated balloons for the treatment of symptomatic intracranial atherosclerosis: initial experience and follow-up outcome. J Neurointervent Surg. (2019) 11:569–73. 10.1136/neurintsurg-2018-014237 30337378

[9] Komotar RJ Starke RM Otten ML Merkow MB Garrett MC Marshall RS. The role of indirect extracranial-intracranial bypass in the treatment of symptomatic intracranial atheroocclusive disease. J Neurosurg. (2009) 110:896–904. 10.3171/2008.9.JNS17658 19199456

[10] Dashti SR Park MS Stiefel MF McDougall CG Albuquerque FC Endovascular recanalization of the subacute to chronically occluded basilar artery: initial experience and technical considerations. Neurosurgery. (2010) 66 4:825–31. 10.1227/01.NEU.0000367611.78898.A3 20190661

[11] He Y Bai W Li T Xue J Wang Z Zhu L. Perioperative complications of recanalization and stenting for symptomatic nonacute vertebrobasilar artery occlusion. Ann Vasc Surg. (2014) 28:386–93. 10.1016/j.avsg.2013.03.014 24200139

[12] Kang DH Kim YW Hwang YH Park SP Kim YS Baik SK. Instant reocclusion following mechanical thrombectomy of *in situ* thromboocclusion and the role of low-dose intra-arterial tirofiban. Cerebrovasc Dis. (2014) 37:350–5. 10.1159/000362435 24941966

[13] Zhao W Che R Shang S Wu C Li C Wu L. Low-dose tirofiban improves functional outcome in acute ischemic stroke patients treated with endovascular thrombectomy. Stroke. (2017) 48:3289–94. 10.1161/STROKEAHA.117.019193 29127270

[14] Yu W Kostanian V Fisher M. Endovascular recanalization of basilar artery occlusion 80 days after symptom onset. Stroke. (2007) 38:1387–9. 10.1161/01.STR.0000260186.93667.a2 17322092

[15] Lin R Aleu A Jankowitz B Kostov D Kanaan H Horowitz M. Endovascular revascularization of chronic symptomatic vertebrobasilar occlusion. J Neuroimaging. (2012) 22:74–9. 10.1111/j.1552-6569.2010.00554.x 21122005

[16] Scheller B Speck U Abramjuk C Bernhardt U Bohm M Nickenig G. Paclitaxel balloon coating, a novel method for prevention and therapy of restenosis. Circulation. (2004) 110:810–4. 10.1161/01.CIR.0000138929.71660.E0 15302790

[17] Tepe G Zeller T Albrecht T Heller S Schwarzwalder U Beregi JP. Local delivery of paclitaxel to inhibit restenosis during angioplasty of the leg. N Engl J Med. (2008) 358:689–99. 10.1056/NEJMoa0706356 18272892

[18] Gruber P Garcia-Esperon C Berberat J Kahles T Hlavica M Anon J. Neuro Elutax SV drug-eluting balloon versus Wingspan stent system in symptomatic intracranial high-grade stenosis: a single-center experience. J Neurointervent Surg. (2018) 10:e32. 10.1136/neurintsurg-2017-013699 29627786

